# hnRNPs in immunity: from nucleic acid sensing to lymphocyte fate determination

**DOI:** 10.3389/fimmu.2026.1958513

**Published:** 2026-09-22

**Authors:** Daiquan Zhang, Jingwen Lei, Shaohui Tang, Shunyan Dong, Xuefei Lei, Lixing Yuan, Xinmei Cao

**Affiliations:** 1 Department of Traditional Chinese Medicine, West China Second University Hospital, Sichuan University, Chengdu, China; 2 Immune Mechanism and Therapy of Major Diseases of Luzhou Key Laboratory, School of Basic Medical Sciences, Southwest Medical University, Luzhou, China; 3 School of nursing, Southwest Medical University, Luzhou, China; 4 Public Laboratory of West China Second University Hospital, Sichuan University, Chengdu, China; 5 Key Laboratory of Birth Defects and Related Diseases of Women and Children (Sichuan University), Ministry of Education, Chengdu, China

**Keywords:** adaptive immunity, alternative splicing, hnRNP, innate immunity, metabolite sensing, nucleic acid sensing, RNA-binding protein

## Abstract

Heterogeneous nuclear ribonucleoproteins (hnRNPs) constitute a family of RNA-binding proteins long recognized for their roles in pre-mRNA processing. However, accumulating evidence has established hnRNPs as central and versatile regulators of immunity. In innate immunity, some hnRNP family members recognize nucleic acids and metabolites to either promote or constrain inflammatory gene expression in a context-dependent manner. In adaptive immunity, they play essential roles in B cell activation and germinal center (GC) responses, as well as T cell activation, survival, and differentiation, primarily through the precise regulation of alternative splicing (AS). Here, we review the functions of hnRNPs (with a focus on A1, A2B1, I, K, L, M, and U) across innate and adaptive immunity, and discuss critical unresolved questions, including the often-overlooked axis of species-specific regulation.

## 1 Introduction

Heterogeneous nuclear ribonucleoproteins (hnRNPs) include approximately 20 RNA-binding proteins with highly modular structural features. They typically have one or more classical RNA-binding domains, including K homology (KH) domains, RNA recognition motifs (RRMs), and arginine-glycine-glycine (RGG) boxes (1). These domains mediate both nonspecific RNA binding and the cooperative recognition of specific RNA secondary structures (1).

hnRNPs modulate transcription, mRNA splicing, nucleocytoplasmic transport, translation, and DNA repair (2–5). The functional versatility of hnRNPs arises from their intrinsic structural disorder and dynamic regulation via post-translational modifications (PTMs), such as phosphorylation and ubiquitination. These modifications rapidly alter hnRNP nucleocytoplasmic shuttling and RNA-binding affinity (2, 6–8). This structural and functional plasticity enables hnRNPs to regulate immune responses.

To prepare this review, we conducted a comprehensive literature search using the keywords “hnRNP”, “innate immunity”, “B cell”, “T cell”, “virus”, and “bacteria” in PubMed and Baidu Scholar, covering publications from the past decade. This review primarily focuses on the roles of hnRNP A1, A2B1, I, K, L, M, and U in both innate and adaptive immunity. hnRNP E1 is briefly discussed where it illustrates specific regulatory principles, but is not the main focus of this review. Additionally, hnRNP F is mentioned in the B cell section as supplementary evidence for specific regulatory principles, but is not a primary focus. In addition, we discuss key unresolved questions and future research directions in this field, with a particular emphasis on the need to disentangle the effects of species, cell type, and pathogen identity on hnRNP function.

## 2 Key functions of hnRNPs in innate immune responses

In the innate immune system, selected hnRNP family members have been shown to serve as direct sensors of pathogen-derived nucleic acids and metabolites, whereas a broader range of hnRNPs contribute to modulating the intensity of inflammatory responses. To provide a clear overview, the key features of hnRNP-mediated innate immune functions are summarized in **Table 1**. Of note, the functional outcomes are highly dependent on the specific combination of organism, cell type, and pathogen, and we have explicitly delineated these axes throughout the text and table.

**Table 1:** Summary of hnRNP-mediated innate immune functions.

hnRNP member	Inducer	Species/cell system	Cellular location	Mechanism	Effect (N/P)	Ref.
hnRNP A1	poly(I:C)	Human A549 lung epithelial cells	Nucleus (basal)/Cytoplasm (upon stress)	Represses basal ISG expression; inhibits IFN-I via suppressing PKR phosphorylation and RNase L body formation, elevating dsRNA threshold	N	(9)
hnRNP A2B1	HSV-1 dsDNA	Mouse MMs/Human THP-1 cells/*Hnrnpa2b1*^fl/fl^ *Lyz2-*Cre mice	Nucleus → Cytoplasm	Activates STING-TBK1-IRF3 pathway	P	(2)
	SFTSV NP/mtDNA	Mouse MEFs/Human THP-1 cells	Cytoplasm	Retains in the cytoplasm via binding NP; Senses mtDNA to activate STING-TBK1 pathway	P	(10)
	LPS/CpG ODNs/Pam3CSK4/*E. coli/S. enterica*/*L. monocytogenes*	Mouse RAW264.7 macrophages/*Hnrnpa2b1*^fl/fl^ *Lyz2-*Cre mice	Nucleus	Metabolite sensing; binds adenine and recruits to *Il1b* enhancers to promote its transcription	P	(11)
		Mouse MMs/*Hnrnpa2b1*^fl/fl^ *LysM-*Cre mice	Nucleus	Regulates the AS of IFN response genes	P	(12)
hnRNP E1	Cytoplasmic ss/dsDNA	Human HEK293 cells	Cytoplasm	Facilitates cGAS-DNA interaction to activate antiviral signal	P	(22)
hnRNP K	*S.* Typhimurium	Mouse RAW264.7 macrophages	Nucleus	Regulates RhoA/Cdc42 signaling through AS	N	(19)
	VSV	Mouse RAW264.7 macrophages	Nucleus	Represses ISG transcription; Regulates AS of genes critical for viral replication	P	(19)
hnRNP M	*L. monocytogenes/*HSV-1/Sendai virus	Human THP-1 cells	Cytoplasm	Forms a complex with ELAVL1/TBK1/IKKϵ/IKKβ/NF-κB p65 to promote IRF3 phosphorylation and type I IFN production	P	(13)
	EMCV/VSV	Human HEK293 cells	Cytoplasm	Blocks viral RNA binding via interaction with RIG-I/MDA5	N	(14)
		Human HMLE mammary epithelial cells	Nucleus	Represses cryptic splicing to prevent endogenous dsRNA formation	N	(15)
hnRNP U	Nuclear dsRNA (HSV-1/VSV)	Mouse iBMDMs/MEFs/DC2.4/Human HEK293/HeLa cells/*Hnrnpu*^fl/fl^*Lyz2*-Cre mice	Nucleus	Oligomerizes and binds to enhancer/super-enhancer of antiviral genes	P	(16)
	SFTSV dsRNA	Mouse MEFs/Human THP-1 cells	Nucleus → Cytoplasm	Activates STING-TBK1 signaling axis following nucleus-to-cytoplasm translocation via interaction with NP	P	(17)
	VSV-induced host RNA	Human THP-1 cells	Nucleus	Increases chromatin accessibility to promote antiviral gene transcription	P	(18)
	VSV	Mouse RAW264.7 cells	Nucleus	Partial loss leads to ISG hyperinduction, restricting VSV replication	N	(19)
	PEDV	Monkey Marc-145 cells	Nucleus → Cytoplasm	Regulates *Traf3* mRNA degradation via METTL3/METTL14/YTHDF2 axis	N	(20)
	IBDV	Chicken DF-1 cells	Nucleus → Cytoplasm	Interacts with MDA5/MAVS to dampen IFN-β expression	N	(21)

hnRNP, heterogeneous nuclear ribonucleoprotein; ISG, interferon-stimulated gene; PKR, protein kinase R; HSV-1, herpes simplex virus-1; dsDNA: double-stranded DNA; MMs, murine macrophages; STING, stimulator of interferon genes; TBK1, tank binding kinase 1; IRF3, interferon regulatory factor 3; N, a negative regulatory role in innate immune responses; P, a positive regulatory role in innate immune responses; SFTSV, severe fever with thrombocytopenia syndrome virus; mtDNA, mitochondrial DNA; MEF, mouse embryonic fibroblast; LPS, lipopolysaccharide; ODNs, oligodeoxynucleotides; *L. monocytogenes*, *Listeria monocytogenes*; *E. coli*, *Escherichia coli*; *S. enterica*, *Salmonella enterica*; AS, alternative splicing; IFN, interferon; cGAS, cyclic GMP-AMP synthase; *S.* Typhimurium, *Salmonella enterica* serovar Typhimurium; VSV, vesicular stomatitis virus; RhoA, Ras homolog family member A; Cdc42, cell division control protein 42; RIG-I, retinoic acid-inducible gene-I; ELAVL1, ELAV-like protein 1; IKKϵ, inhibitor of kappa B kinase-ϵ; NF-κB, nuclear factor kappa-B; EMCV, encephalomyocarditis virus; iBMDMs, immortalized bone marrow-derived macrophages; PEDV, porcine epidemic diarrhea virus; *Traf3*, TNF receptor associated factor 3; METTL3, methyltransferase-like protein 3; YTHDF2, YTH N6-methyladenosine RNA binding protein 2; IBDV, infectious bursal disease virus; MDA5, melanoma differentiation-associated gene 5; MAVS, mitochondrial antiviral signaling protein.

### 2.1 hnRNP A1

Deshaies et al. demonstrated that hnRNP A1 represses the basal expression of interferon-stimulated genes (ISGs), including multiple members of the oligoadenylate synthase family (key dsRNA sensors), thereby establishing a higher threshold for dsRNA-triggered antiviral responses in human A549 lung epithelial cells (9). Upon poly(I:C) treatment, nuclear hnRNP A1 translocates to the cytoplasm and is partially recruited to RNase L bodies (9). siRNA-mediated knockdown of *HNRNPA1* in A549 cells enhances protein kinase R phosphorylation, accelerates RNase L body formation, and potentiates type I interferon production upon poly(I:C) stimulation (9).

### 2.2 hnRNP A2B1

#### 2.2.1 Senses nucleic-acids and positively regulates anti-viral innate immunity

Wang et al. demonstrated that, upon DNA virus infection (e.g., herpes simplex virus, HSV-1), *Hnrnpa2b1* knockout in murine macrophages (including RAW 264.7 cells, bone marrow-derived macrophages (BMDMs), and primary peritoneal macrophages), as well as its knockdown in human THP-1 cells (a monocytic cell line), reduces the expression of IFN-α and IFN-β, whereas the production of TNF-α and IL-6 remains largely unaffected. Consistently, *Hnrnpa2b1*^fl/fl^*Lyz2-*Cre mice exhibit lower IFN-β levels compared with control littermates. Mechanistically, nuclear hnRNP A2B1 directly binds viral double-stranded DNA (dsDNA), undergoes homodimerization, and translocates to the cytoplasm, where it activates the STING-TBK1-IRF3 signaling axis to stimulate type I interferon production. Furthermore, hnRNP A2B1 facilitates N^6^-methyladenosine modification and the nucleocytoplasmic trafficking of mRNA (including those encoding cGAS, STING, and interferon-inducible protein 16) to enhance IFN-β pathway activation (2).

Interestingly, the antiviral mechanism of hnRNP A2B1 appears to be pathogen-context-dependent. Following infection with severe fever with thrombocytopenia syndrome virus (SFTSV, an RNA virus), the viral nucleocapsid protein (NP) interacts with hnRNP A2B1 and retains it in the cytoplasm, facilitating its recognition of mislocalized mitochondrial DNA and subsequent activation of the STING-TBK1 axis. In this setting, CRISPR/Cas9-mediated *Hnrnpa2b1* knockout in mouse embryonic fibroblasts (MEFs) and human THP-1 cells reduces the production of IFN-β, IL-1β, and IL-6 (10).

Collectively, these findings indicate that hnRNP A2B1 orchestrates antiviral responses through distinct molecular strategies depending on the nature of the invading virus, and that this function is conserved across human and mouse systems, at least at the phenotypic level.

#### 2.2.2 As a metabolite sensor

Distinct from its role as a noncanonical nucleic-acid sensor, hnRNP A2B1 also functions as a nuclear sensor for the endogenous metabolite adenine. Zhang et al. found that nuclear adenine levels in mouse macrophages increase sharply following bacterial infection or stimulation with lipopolysaccharide (LPS), CpG oligodeoxynucleotides, or Pam3CSK4. Upon directly sensing elevated nuclear adenine, hnRNP A2B1 is recruited to interleukin-1 beta (*Il1b*) enhancers to induce IL-1β expression. This response protects mice from lethal infection with *Escherichia coli* or *Listeria monocytogenes* (*L. monocytogenes*), as *Hnrnpa2b1*^fl/fl^*Lyz2-*Cre mice exhibit significantly lower survival rates (11). Collectively, these findings illustrate that hnRNPs can sense host-derived metabolites as danger signals to initiate antibacterial immune responses, thereby extending the concept of innate immune sensing beyond classical pathogen-associated molecular pattern (PAMP) recognition.

#### 2.2.3 Regulates alternative splicing

Salih et al. demonstrated that myeloid-specific conditional ablation of *Hnrnpa2b1* in mice leads to attenuated inflammatory responses and increased susceptibility to *Salmonella enterica* infection. This phenotype is attributed to the role of hnRNP A2B1 in regulating the AS of specific immune genes (including *Ifngr1*, *Stat1*, *Stat3*, *Oas3*, and *Irf7*) that are critical for macrophage responsiveness to IFN-γ (12). Additionally, RNA-seq analysis of *Hnrnpa2b1*-deficient BMDMs stimulated with LPS revealed that genes involved in cell division and DNA replication are also upregulated (12), which indicates that hnRNP A2B1 exerts multiple functions during inflammation beyond its canonical role in splicing regulation.

### 2.3 hnRNP M

hnRNP M plays dual roles in the regulation of innate immunity, exerting both positive and negative effects depending on the cellular context and mechanism involved.

#### 2.3.1 Promotes anti-pathogen innate immunity

Kirchhoff et al. identified that depletion of hnRNP M in human THP-1 cells reduces type-I IFN induction in response to *L. monocytogenes*, HSV-1, and Sendai virus infection. Mechanistically, hnRNP M forms a complex with ELAV-like protein 1 (ELAVL1). This complex positively modulates the formation of a multiprotein complex containing IKKϵ, IKKβ, TBK1, and NF-κB p65 downstream of both RIG-I and cGAS, which promotes IRF3 phosphorylation and type I interferon induction (13). These findings establish hnRNP M as a positive regulator of anti-pathogen innate immunity in human myeloid cells.

#### 2.3.2 Suppresses innate immune responses

hnRNP M also suppresses innate immunity via two distinct mechanisms. Cao et al. reported that in human HEK293 cells infected with encephalomyocarditis virus (EMCV) or VSV, hnRNP M directly interacts with RIG-I and melanoma differentiation-associated gene 5 (MDA5). This interaction blocks the binding of RIG-I and MDA5 to viral RNA, consequently suppressing innate immune activation at the pathogen recognition stage (14). This inhibitory function may limit excessive antiviral immune responses and prevent associated immunopathology. Zheng et al. revealed an alternative inhibitory mechanism. Using individual-nucleotide resolution UV crosslinking and immunoprecipitation (iCLIP) analysis, they identified 54,377 high-confidence hnRNP M-binding sites in human mammary epithelial cells, the vast majority of which reside in deep intronic regions. In addition, hnRNP M represses cryptic splicing events arising from intronic long interspersed nuclear elements, which prevents the formation of endogenous dsRNA that would trigger type I interferon responses. Notably, *HNRNPM*-knockdown human HMLE mammary epithelial cells exhibit elevated interferon signaling, and tumors with low hnRNP M expression, including breast cancer, are associated with improved patient prognosis (15), suggesting that hnRNP M may represent a potential therapeutic target for cancer immunotherapy.

Collectively, the opposing regulatory effects of hnRNP M on innate immunity are context-dependent, varying with cell type, infecting pathogen, and the specific step of the immune signaling cascade involved. Notably, the positive regulatory role has been demonstrated in human THP-1 cells, while the negative roles have been observed in human HEK293 and HMLE cells, suggesting that cell type, rather than species, may be the primary determinant of hnRNP M’s dual functions in humans.

### 2.4 hnRNP U

#### 2.4.1 Senses RNA and positively regulates antiviral innate immunity

hnRNP U (also known as scaffold attachment factor A, SAFA) senses viral dsRNA in both the nucleus and the cytoplasm through distinct mechanisms. Cao et al. found that INF-β production is markedly reduced in *Hnrnpu*^-/-^ cells (including mouse immortalized bone marrow-derived macrophages (iBMDM), MEFs, DC2.4, and human HeLa and HEK293 cells) following HSV-1 or VSV infection, and that hnRNP U reconstitution restores INF-β expression. In line with these *in vitro* findings, *Hnrnpu*^fl/fl^*Lyz2*-Cre mice infected with either virus display enhanced susceptibility due to impaired type I IFN production. These data indicate that hnRNP U is critical for antiviral immunity across human and mouse system (16). Mechanistically, immunoprecipitation coupled with RNA immunoprecipitation sequencing (RIP-seq) reveals that hnRNP U directly binds to nuclear dsRNA (derived from both HSV-1 and VSV) via its RGG domain. This binding event induces hnRNP U oligomerization and subsequently activates antiviral gene transcription (including *Ifnb1*) through binding to enhancers and super-enhancers (16). Additionally, hnRNP U senses cytoplasmic viral dsRNA. During severe fever with thrombocytopenia syndrome virus (SFTSV) infection, hnRNP U interacts with the viral NP via its nuclear localization signal domain. This interaction drives its translocation from the nucleus to the cytoplasm. Once in the cytoplasm, hnRNP U binds viral dsRNA and activates the STING-TBK1 signaling axis, thereby initiating antiviral responses. Concordantly, *HNRNPU* knockdown in human THP-1 cells, as well as knockout in both human THP-1 and mouse MEF cells, attenuates type I IFN production and promotes viral propagation (17). In addition to these direct sensing pathways, hnRNP U also contributes to an indirect surveillance mechanism. This mechanism involves the recognition of virus-induced host RNA. In the human THP-1 cells infected with VSV for 24 hours, hnRNP U interacts with virus-induced host RNA. This binding increases chromatin accessibility at immune gene loci and enhances antiviral gene transcription (18). An intriguing and fundamentally important question that remains to be addressed is how hnRNP U discriminates between viral RNA and host-derived RNA to selectively trigger antiviral immunity while avoiding autoimmunity.

#### 2.4.2 Negatively regulates antiviral innate immunity

Intriguingly, hnRNP U can function as either a positive or negative regulator of antiviral innate immunity depending on the cellular environment and species. Whereas hnRNP U exerts an antiviral effect in VSV-infected human THP-1 cells, its knockdown in mouse RAW264.7 macrophages inhibits VSV replication at 4–12 hours post-infection, an effect attributable to the spontaneous hyperinduction of a cluster of ISGs (including *Irf7*, *Isg15*, *Ifi44l*, and *Trex1*) even in the absence of infection. This pre-existing antiviral transcriptional state renders the cells highly refractory to subsequent viral infection. Notably, this phenotype arises from changes in steady-state transcript abundance rather than from widespread AS events (19). In monkey Marc-145 cells, Zhou et al. found that hnRNP U overexpression promotes porcine epidemic diarrhea virus (PEDV) replication, whereas its knockdown inhibits viral replication. Mechanistically, PEDV infection triggers the cytoplasmic translocation of nuclear hnRNP U, which enhances m^6^A-methylation of TNF receptor associated factor 3 (*Traf3*) mRNA via the methyltransferase-like protein 3 (METTL3)-METTL14/YTH N6-methyladenosine RNA binding protein 2 (YTHDF2) signaling axis. Consequently, this modification promotes the decay of *Traf3* transcripts, which impairs the expression of IFN-β and downstream antiviral genes, ultimately facilitating PEDV replication (20). Similarly, in chicken DF-1 cells, hnRNP U interacts with MDA5 and mitochondrial antiviral signaling protein (MAVS) to dampen MDA5/MAVS-mediated IFN-β induction, and its overexpression promotes infectious bursal disease virus replication (21).

Collectively, hnRNP U exerts dual regulatory effects on antiviral innate immunity. The net outcome is highly dependent on species, cell type, pathogen, and the timing of infection, highlighting the complexity of its immunomodulatory roles. For instance, hnRNP U is positively regulatory in human THP-1 and mouse iBMDMs/MEFs but negatively regulatory in mouse RAW264.7, monkey Marc-145, and chicken DF-1 cells—systems that differ not only in species but also in cell type and host-pathogen pairing.

### 2.5 Other hnRNP family members in innate immune regulation

Using deletion and immunoprecipitation assays, Liao et al. demonstrated that hnRNP E1, or poly(rC)-binding protein 1 (PCBP1), directly recognizes cytoplasmic single-stranded DNA (ssDNA) or dsDNA through its KH1 domain in human HEK293 cells, which thereby facilitates cGAS engagement with these nucleic acids and initiates antiviral innate immune responses (22).

Wagner et al. demonstrated that in mouse RAW264.7 macrophages, *Hnrnpk* knockdown renders cells resistant to VSV replication. This antiviral effect arises from two interconnected consequences: first, the spontaneous upregulation of a set of ISGs due to relief of hnRNP K-mediated transcriptional repression; and second, hnRNP K-dependent AS of host genes important for viral replication (e.g., *Gpbp1*, *Eri2*, *Setd5*, and *Arhgap12*). Conversely, in the same RAW264.7 knockdown system, *Hnrnpk* knockdown promotes *Salmonella enterica* serovar Typhimurium (*S.* Typhimurium) replication by regulating AS of genes involved in RhoA and Cdc42 signaling, which are necessary for bacterial entry and intracellular survival (19). Collectively, hnRNP K functions as a host factor that restricts viral replication yet supports antibacterial control in the same cell type, likely through splicing-mediated regulation of distinct gene sets tailored to each pathogen.

Together, these observations demonstrate that hnRNPs exert either pro- or anti-inflammatory immune activities depending on the pathogen type, cellular context, and infection stage.

## 3 Core roles of hnRNPs in adaptive immune responses

The precise regulation of gene splicing by hnRNPs plays pivotal roles in adaptive immune cells, particularly in governing the activation, differentiation, and effector functions of B and T lymphocytes.

### 3.1 Regulation of B cell activation and germinal center responses

In the LPS-activated mouse pro-B cell line 38B9, hnRNP A2B1 binds to major histocompatibility complex (MHC) class II molecules. This binding regulates mitogen-activated protein kinase signaling, protein kinase B (also known as AKT) activation, and the NF-κB pathways, thereby promoting B cell maturation and differentiation (23). Furthermore, in mouse B cells, hnRNP A2B1 and hnRNP A1 regulate the AS of zeste homolog 2 (*Ezh2*). They interact with UAG-rich motifs within intron 14 of the *Ezh2* pre-mRNA to induce exon 14 inclusion. This splicing event maintains EZH2 enzymatic activity. Consequently, it represses *Cdkn1a* (encoding p21, a cyclin-dependent kinase inhibitor) and facilitates early B cell proliferation and GC initiation (24). In contrast, hnRNP A2B1 and hnRNP A1 bind to G-tracts in *Cd40* intron 6. This binding promotes skipping of *Cd40* exon 6, which encodes the CD40 transmembrane domain. Consequently, this event impairs surface CD40 expression and compromises GC maintenance (4). In this context, hnRNP F is mentioned as supplementary evidence supporting the critical role of RNA-binding proteins in regulating GC responses, although it is not a primary focus of this review. Although these findings appear paradoxical, they may be reconciled by the distinct target genes and the differential binding site preference at specific stages of B cell differentiation. This reflects the context-dependent regulatory functions of hnRNP A1 and hnRNP A2B1 during the GC response.

Polypyrimidine tract-binding protein 1 (PTBP1, or hnRNP I) and its compensatory paralog PTBP2 are necessary for B cell development, as their combined deletion completely blocks B cell ontogeny at the pro-B cell stage in mice (25). Mechanistically, PTBP1 orchestrates a post-transcriptional mRNA regulon that precisely regulates CDK activity across the cell cycle—repressing S-phase entry while promoting G2/M transition—thereby ensuring proper progression through the critical proliferative stages of early B lymphopoiesis (25). In the GC, PTBP1 is upregulated in positively selected B cells upon T cell help and controls both mRNA abundance and AS of key c-MYC-dependent genes that are critical for metabolic reprogramming and nucleotide synthesis during B cell proliferation. Consequently, *Ptbp1*^fl/fl^*Cd79a-*cre GC B cells exhibit defective late S-phase progression, increased apoptosis in the dark zone, and attenuated c-MYC-driven transcriptional programs in response to positive selection in the light zone (26). Thus, PTBP1 regulates both early B-lymphopoiesis and GC responses through fine-tuning CDK activity and c-MYC-dependent transcriptional programs.

hnRNP K plays a critical role in antibody class switch recombination (CSR) and somatic hypermutation (SHM) induced by activation-induced deaminase (AID). In mouse CH12F3-2A cells and human BL2 cells, hnRNP K interacts with AID monomers through its RNA-binding motifs (including RGG and GXXG) in an RNA-dependent manner. Mutation of these motifs diminishes the nuclear retention of both hnRNP K and AID and impairs AID-mediated DNA breaks. Accordingly, siRNA-mediated *Hnrnpk* knockdown reduces IgA class switching in CH12F3-2A cells and compromises SHM in BL2 cells, without affecting AID expression or germline transcripts (GLTs, which are essential for CSR) (27–29).

hnRNP L is essential for B cell activation and CSR in mice. *Hnrnpl*^fl/fl^*Cd21*-cre mice exhibit reduced marginal-zone B cell populations and diminished T cell-dependent antibody responses (3). In IL-4/LPS-activated B cells isolated from *Rosa*^mT/mG^*Hnrnpl*^fl/fl^ *Cd21*-cre mice, hnRNP L deficiency leads to proliferation defects and enhanced apoptosis. These defects are attributable to its regulation of AS of epigenetic modifiers such as sirtuin 1 (*Sirt1*) and nuclear receptor-binding SET domain protein 2 (*Nsd2*), which disrupts MYC- and E2F-driven transcription and impairs mitochondrial function, resulting in increased reactive oxygen species (3). Furthermore, hnRNP L regulates CSR through multiple mechanisms. It binds AID dimers in an RNA-dependent manner, and its deficiency reduces post-cleavage recombination and impairs CSR in CH12F3-2A cells, without affecting SHM in BL2 cells (28). Mechanistically, hnRNP L forms a complex with cleavage stimulating factor 64 to recruit 53BP1 and Ku80, thereby promoting post-cleavage DNA repair via NHEJ pathway (30). Additionally, hnRNP L binds to *IgH* switch regions—predominantly to the Sμ sterile GLT region in resting and LPS/IL-4-activated splenic B cells, and specifically to the Sγ1 GLT upon activation. Compared with control cells from *Rosa*^mT/mG^*Hnrnpl*^fl/+^*Cd21*-cre mice, *Hnrnpl-*deficient B cells exhibit reduced Sγ1 GLT levels, further contributing to CSR impairment under LPS/IL-4 stimulation (3).

hnRNP U also associates with AID via RNA (29, 31) and supports CSR by promoting liquid-liquid phase separation, which recruits Ku80 and 53BP1 to DNA break sites within the *IgH* switch regions, while simultaneously preventing R-loop accumulation and ssDNA formation via its interaction with G-quadruplex structures in switch-region RNA/DNA (32).

### 3.2 Regulation of T cell activation, survival, and differentiation

PTBP1 is an important regulator of CD4^+^ T cell activation, as its knockdown in human CD4^+^ T cells impairs proliferation and reduces the expression of activation-triggered molecules, including CD25, CD69, CD40L, IL-2, and TNF-α (33). Mechanistically, PTBP1 modulates the stability of *CD40L* and *IL2* transcripts via activation-dependent mRNA decay, and influences early TCR signaling through the ERK1/2 and NF-κB pathways (33). PTBP1 is also necessary for CD8^+^ T cell activation. D’Angeli et al. demonstrated that conditional deletion of *Ptbp1* in mouse CD8^+^ T cells does not affect naive T cell development, but is indispensable for optimal activation, proliferation, and survival following antigen stimulation. At the molecular level, PTBP1 controls the AS of *Ppp3cb* (encoding the calcineurin Aβ catalytic subunit) to promote the expression of specific isoforms that facilitate c-Fos expression and the nuclear accumulation of nuclear factor of activated T cells 2 (NFATc2) and NFATc3, thereby ensuring proper IL-2 and TNF-α production and effector differentiation (34). Notably, the AS target *Ppp3cb* in mouse T cells is the ortholog of *PPP3CB* in humans, suggesting potential conservation of this regulatory axis, although direct evidence in human T cells is lacking.

hnRNP L governs multiple aspects of T cell biology (including development, activation, survival, proliferation, and differentiation), primarily through the regulation of AS and mRNA metabolism. Through cross-linking and immunoprecipitation followed by high throughput sequencing, Shankarling et al. revealed that hnRNP L binds to and modulates the AS of genes important for TCR and Wnt signaling in both human Jurkat and primary human CD4^+^ T cells (35). Gaudreau et al. deleted hnRNP L in T cells using *Lck*-Cre mice, which leads to a thymic developmental block at the double-negative 4-to-double-positive transition and failure of single-positive T cells to reach the periphery. This phenotype is attributed to aberrant *Cd45ra* splicing, altered LCK phosphorylation (with increased phosphorylation at Y394 rather than Y505), and mis-splicing of genes encoding cytoskeletal and GTPase regulators (36), underscoring the essential role of hnRNP L in thymocyte maturation and migration. In contrast, White et al. generated *Hnrnpl*^fl/fl^*Cd4*-cre mice, in which Cre is expressed at the double-positive stage or later, thus sparing early thymic development. In this model, hnRNP L deletion does not perturb thymic development but markedly reduces peripheral T cell numbers and impairs differentiation into Th1, Th2, and Treg subsets *in vitro*, as well as Tfh-mediated GC responses *in vivo* (37). Mechanistically, activated hnRNP L-deficient T cells undergo caspase-dependent apoptosis due to a failure to upregulate BCL-XL (37). Furthermore, hnRNP L serves as a direct substrate of AKT during Treg induction under low-dose TCR stimulation. Its phosphorylation by AKT orchestrates splicing programs that favor Treg differentiation, as hnRNP L knockdown impairs Treg induction and promotes Th cell generation (8). Collectively, these studies establish hnRNP L as a key post-transcriptional regulator that integrates TCR signal strength with splicing programs to control T cell fate, survival, and effector function, while also underscoring that the developmental stage of deletion critically determines whether the primary defect lies in thymic selection or peripheral homeostasis.

## 4 Conclusion and perspectives

hnRNPs have emerged as central hubs within immune regulatory networks, orchestrating diverse biological processes spanning nucleic acid and metabolite sensing, transcriptional modulation, and AS-dependent gene control. In innate immunity, certain hnRNPs directly recognize pathogen-derived molecules, with some family members promoting inflammation while others exert suppressive functions. In adaptive immunity, hnRNPs profoundly influence lymphocyte fate through the AS of key genes governing B cell activation, GC responses, as well as T cell activation, differentiation, and survival.

Despite substantial progress, several critical questions remain to be addressed. First, much of the current data on hnRNP functions derive from *in vitro* experiments and thus require rigorous validation using cell-type-specific knockout models *in vivo*. Second, while most studies have focused on AS regulation, the functional impact of post-translational modifications (PTMs) on hnRNP activity in immune settings remains largely underexplored. Third, and perhaps most critically, the field must move beyond treating species, cell type, and pathogen as a single “context” variable. Our analysis of the literature, particularly for hnRNP U, reveals starkly different outcomes across human, mouse, monkey, and chicken systems. It remains largely unknown whether hnRNPs regulate the same AS events and target genes across species, or whether their functions have diverged. Future studies should employ cross-species comparative approaches, such as CLIP-seq in matched cell types from different organisms, to systematically address this fundamental question. Fourth, given that hnRNPs govern fundamental cellular processes (including RNA processing, cell proliferation, survival, and DNA repair), global inhibition of a given hnRNP may cause substantial toxicity. Consequently, more realistic therapeutic strategies might involve targeting specific isoforms, interaction domains, PTMs, or aberrant subcellular localization, rather than eliciting broad suppression. Addressing these questions will deepen our understanding of hnRNP biology and may facilitate the development of innovative immunomodulatory strategies for infections, inflammation, and malignancies.

## References

[1] Sudhakaran M Doseff AI . Role of heterogeneous nuclear ribonucleoproteins in the cancer-immune landscape. Int J Mol Sci. (2023) 24:5086. doi: 10.3390/ijms24065086 36982162 PMC10049280

[2] Wang L Wen M Cao X . Nuclear hnRNPA2B1 initiates and amplifies the innate immune response to DNA viruses. Science. (2019) 365:eaav0758. doi: 10.1126/science.aav0758 31320558

[3] Subramani PG Fraszczak J Helness A Estall JL Moroy T Di Noia JM . Conserved role of hnRNPL in alternative splicing of epigenetic modifiers enables B cell activation. EMBO Rep. (2024) 25:2662–97. doi: 10.1038/s44319-024-00152-3 38744970 PMC11169469

[4] Huang H Li Y Zhang G Ruan GX Zhu Z Chen W . The RNA-binding protein hnRNP F is required for the germinal center B cell response. Nat Commun. (2023) 14:1731. doi: 10.1038/s41467-023-37308-z 36997512 PMC10063658

[5] Klaric JA Wust S Panier S . New faces of old friends: Emerging new roles of RNA-binding proteins in the DNA double-strand break response. Front Mol Biosci. (2021) 8:668821. doi: 10.3389/fmolb.2021.668821 34026839 PMC8138124

[6] Britton S Froment C Frit P Monsarrat B Salles B Calsou P . Cell nonhomologous end joining capacity controls SAF-A phosphorylation by DNA-PK in response to DNA double-strand breaks inducers. Cell Cycle. (2009) 8:3717–22. doi: 10.4161/cc.8.22.10025 19844162

[7] Herrmann C Dybas JM Liddle JC Price AM Hayer KE Lauman R . Adenovirus-mediated ubiquitination alters protein-RNA binding and aids viral RNA processing. Nat Microbiol. (2020) 5:1217–31. doi: 10.1038/s41564-020-0750-9 32661314 PMC7529849

[8] Hawse WF Boggess WC Morel PA . TCR signal strength regulates Akt substrate specificity to induce alternate murine Th and T regulatory cell differentiation programs. J Immunol. (2017) 199:589–97. doi: 10.4049/jimmunol.1700369 28600288 PMC5575766

[9] Deshaies JE Triassi V Lacombe A Gagne M Ling K Ghosh A . The differential impact of HNRNPA1 isoforms on gene expression and their relevance to dsRNA-mediated innate immune response. Sci Rep. (2025) 15:15306. doi: 10.1038/s41598-025-99031-7 40312500 PMC12046027

[10] Huang X Zhang Y Fan J Yu X Fang Y Ren J . hnRNPA2B1 recognizes RNA virus SFTSV infection through mitochondrial DNA. mBio. (2025) 16:e0166825. doi: 10.1128/mbio.01668-25 40689679 PMC12345236

[11] Zhang S Cui Z Zhang D Zhang D Jin K Li Z . Nuclear adenine activates hnRNPA2B1 to enhance antibacterial innate immunity. Cell Metab. (2025) 37:413–28. doi: 10.1016/j.cmet.2024.11.014 39814017

[12] Salih MM Weindel CG Malekos E Sudek L Katzman S Mabry CJ . Myeloid-specific HNRNPA2B1 deficiency disrupts macrophage function and *in vivo* responses. J Immunol. (2025) 214:2041–54. doi: 10.1093/jimmun/vkaf073 40414614 PMC12394992

[13] Kirchhoff A Herzner AM Urban C Piras A Duster R Mahlberg J . RNA-binding proteins hnRNPM and ELAVL1 promote type-I interferon induction downstream of the nucleic acid sensors cGAS and RIG-I. EMBO J. (2025) 44:824–53. doi: 10.1038/s44318-024-00331-x 39707025 PMC11791083

[14] Cao P Luo WW Li C Tong Z Zheng ZQ Zhou L . The heterogeneous nuclear ribonucleoprotein hnRNPM inhibits RNA virus-triggered innate immunity by antagonizing RNA sensing of RIG-I-like receptors. PloS Pathog. (2019) 15:e1007983. doi: 10.1371/journal.ppat.1007983 31433824 PMC6703689

[15] Zheng R Dunlap M Bobkov GOM Gonzalez-Figueroa C Patel KJ Lyu J . hnRNPM protects against the dsRNA-mediated interferon response by repressing LINE-associated cryptic splicing. Mol Cell. (2024) 84:2087–103. doi: 10.1016/j.molcel.2024.05.004 38815579 PMC11204102

[16] Cao L Liu S Li Y Yang G Luo Y Li S . The nuclear matrix protein SAFA surveils viral RNA and facilitates immunity by activating antiviral enhancers and super-enhancers. Cell Host Microbe. (2019) 26:369–84. doi: 10.1016/j.chom.2019.08.010 31513772

[17] Liu BY Yu XJ Zhou CM . SAFA initiates innate immunity against cytoplasmic RNA virus SFTSV infection. PloS Pathog. (2021) 17:e1010070. doi: 10.1371/journal.ppat.1010070 34788350 PMC8598041

[18] Cao L Luo Y Guo X Liu S Li S Li J . SAFA facilitates chromatin opening of immune genes through interacting with anti-viral host RNAs. PloS Pathog. (2022) 18:e1010599. doi: 10.1371/journal.ppat.1010599 35658050 PMC9200321

[19] Wagner AR Scott HM West KO Vail KJ Fitzsimons TC Coleman AK . Global transcriptomics uncovers distinct contributions from splicing regulatory proteins to the macrophage innate immune response. Front Immunol. (2021) 12:656885. doi: 10.3389/fimmu.2021.656885 34305890 PMC8299563

[20] Zhou H Yan Y Gao J Ma M Liu Y Shi X . Heterogeneous nuclear protein U degraded the m(6)A methylated TRAF3 transcript by YTHDF2 to promote porcine epidemic diarrhea virus replication. J Virol. (2023) 97:e0175122. doi: 10.1128/jvi.01751-22 36752613 PMC9973030

[21] Hu X Wu X Ding Z Chen Z Wu H . Characterization and functional analysis of chicken dsRNA binding protein hnRNPU. Dev Comp Immunol. (2023) 138:104521. doi: 10.1016/j.dci.2022.104521 36044969

[22] Liao CY Lei CQ Shu HB . PCBP1 modulates the innate immune response by facilitating the binding of cGAS to DNA. Cell Mol Immunol. (2021) 18:2334–43. doi: 10.1038/s41423-020-0462-3 32415261 PMC8484664

[23] Park J Choe CH Kim J Yang JS Kim JH Jang H . Heterogeneous nuclear ribonucleoprotein A2B1 exerts a regulatory role in lipopolysaccharide-stimulated 38B9 B cell activation. Immune Netw. (2017) 17:437–50. doi: 10.4110/in.2017.17.6.437 29302256 PMC5746613

[24] Zhu Z Zhang R Kang H Chen M Huang H Wang J . HnRNP A1 and A2B1 enforce Ezh2 mRNA splicing to promote germinal center B cell responses. Nat Commun. (2026) 17:7436. doi: 10.1038/s41467-026-74159-w 42277033 PMC13408116

[25] Monzon-Casanova E Matheson LS Tabbada K Zarnack K Smith CW Turner M . Polypyrimidine tract-binding proteins are essential for B cell development. Elife. (2020) 9:e53557. doi: 10.7554/elife.53557 32081131 PMC7058386

[26] Monzon-Casanova E Screen M Diaz-Munoz MD Coulson RMR Bell SE Lamers G . The RNA-binding protein PTBP1 is necessary for B cell selection in germinal centers. Nat Immunol. (2018) 19:267–78. doi: 10.1038/s41590-017-0035-5 29358707 PMC5842895

[27] Yin Z Kobayashi M Hu W Higashi K Begum NA Kurokawa K . RNA-binding motifs of hnRNP K are critical for induction of antibody diversification by activation-induced cytidine deaminase. Proc Natl Acad Sci USA. (2020) 117:11624–35. doi: 10.1073/pnas.1921115117 32385154 PMC7261133

[28] Hu W Begum NA Mondal S Stanlie A Honjo T . Identification of DNA cleavage- and recombination-specific hnRNP cofactors for activation-induced cytidine deaminase. Proc Natl Acad Sci USA. (2015) 112:5791–6. doi: 10.1073/pnas.1506167112 25902538 PMC4426456

[29] Mondal S Begum NA Hu W Honjo T . Functional requirements of AID's higher order structures and their interaction with RNA-binding proteins. Proc Natl Acad Sci USA. (2016) 113:E1545–1554. doi: 10.1073/pnas.1601678113 26929374 PMC4801308

[30] Haque F Nakata M Kosako H Honjo T Begum NA . hnRNPL-CstF64 complex: Coordinating CSR and LSR in IgH locus recombination dynamics through eRNA and NHEJ regulation. Nucleic Acids Res. (2025) 53. doi: 10.1093/nar/gkaf810 40902003 PMC12407099

[31] Al Ismail A Husain A Kobayashi M Honjo T Begum NA . Depletion of recombination-specific cofactors by the C-terminal mutant of the activation-induced cytidine deaminase causes the dominant negative effect on class switch recombination. Int Immunol. (2017) 29:525–37. doi: 10.1093/intimm/dxx061 29136157

[32] Refaat AM Nakata M Husain A Kosako H Honjo T Begum NA . HNRNPU facilitates antibody class-switch recombination through C-NHEJ promotion and R-loop suppression. Cell Rep. (2023) 42:112284. doi: 10.1016/j.celrep.2023.112284 36943867

[33] La Porta J Matus-Nicodemos R Valentin-Acevedo A Covey LR . The RNA-binding protein, polypyrimidine tract-binding protein 1 (PTBP1) is a key regulator of CD4 T cell activation. PloS One. (2016) 11:e0158708. doi: 10.1371/journal.pone.0158708 27513449 PMC4981342

[34] D'Angeli V Monzon-Casanova E Matheson LS Gizlenci O Petkau G Gooding C . Polypyrimidine tract binding protein 1 regulates the activation of mouse CD8 T cells. Eur J Immunol. (2022) 52:1058–68. doi: 10.1002/eji.202149781 PMC9546061 35460072

[35] Shankarling G Cole BS Mallory MJ Lynch KW . Transcriptome-wide RNA interaction profiling reveals physical and functional targets of hnRNP L in human T cells. Mol Cell Biol. (2014) 34:71–83. doi: 10.1128/mcb.00740-13 24164894 PMC3911283

[36] Gaudreau MC Heyd F Bastien R Wilhelm B Moroy T . Alternative splicing controlled by heterogeneous nuclear ribonucleoprotein L regulates development, proliferation, and migration of thymic pre-T cells. J Immunol. (2012) 188:5377–88. doi: 10.4049/jimmunol.1103142 22523384

[37] White TLA Henry DJ Roberts SDA Jin Y Gong YN Morel PA . HnRNP L is essential for peripheral T cell proliferation and survival. Front Immunol. (2025) 16:1543145. doi: 10.3389/fimmu.2025.1543145 40276511 PMC12018431

